# Bridging Perspectives, Building Resilience: Safety-II Guided Reflexive Dialogues Between Care Professionals and Clients as Part of Developing Integrated Maternity Care

**DOI:** 10.5334/ijic.8588

**Published:** 2024-10-22

**Authors:** Sarah R. Lips, Jolanda C. G. Boxem-Tiemessen, Anna M. Ligthart, Tjerk Jan Schuitmaker-Warnaar, Martine C. de Bruijne, Corine J. M. Verhoeven, Petra Verdonk, Ank de Jonge

**Affiliations:** 1 Amsterdam UMC location Vrije Universiteit Amsterdam, Department of Ethics, Law & Humanities, Amsterdam, The Netherlands; 2 Amsterdam Public Health, Quality of Care, Amsterdam, The Netherlands; 3 Amsterdam UMC location Vrije Universiteit Amsterdam, Department of Midwifery Science, Amsterdam, The Netherlands; 4 Midwifery Academy Amsterdam Groningen, InHolland, Amsterdam, The Netherlands; 5 University of Groningen, University Medical Center Groningen, Department of Primary and Long-Term Care, Groningen, The Netherlands; 6 Independent client of VSV Westfriesland-Waterland/IGO Geboortehart, The Netherlands; 7 Athena Institute, Vrije Universiteit Amsterdam, Amsterdam, The Netherlands; 8 Amsterdam UMC location Vrije Universiteit Amsterdam, Department of Public and Occupational Health, Amsterdam, The Netherlands; 9 Division of Midwifery, School of Health Sciences, University of Nottingham, Nottingham, United Kingdom; 10 Department of Obstetrics and Gynaecology, Maxima Medical Centre, Veldhoven, The Netherlands

**Keywords:** integrated maternity care, participatory action research, reflexivity, dialogue, resilience, relation-centered care, Safety-II

## Abstract

**Background:**

Limitations of traditional structures and approaches to further enhance patient safety, satisfaction, and systemic sustainability in healthcare, are becoming increasingly visible. Embedding reflexivity is a proposed strategy to promote progress. We aimed to explore the potential of creating reflexive spaces for promoting integration and client-centeredness in maternity care specifically.

**Methods:**

In this participatory action research (PAR), two multidisciplinary and multiorganizational groups of maternity care professionals and clients (n = 28) from two Dutch regions, participated in ‘reflexive dialogues’. Cases were discussed from a Safety-II perspective. In total, 22 meetings took place from 2020–2022, mostly online. Additionally, 23 participants were interviewed. Data were audio-recorded, transcribed, and thematically analyzed.

**Findings:**

Participants were generally positive about the reflexive dialogues and Safety-II approach. They felt both safe and challenged to critically reflect on their own and each other’s care practices. Exchanging perspectives, experiences, and approaches fostered trust, well-being, and repertoire, and through this, resilience.

**Conclusions:**

By structurally stimulating, facilitating, and embedding Safety-II guided reflexive dialogues between professionals and clients from multiple organizations and disciplines, healthcare leaders could promote resilience and reinforce the transformation towards integrated, relation-centered maternity care.

## Introduction

Limitations of traditional structures and approaches in the healthcare system, to further enhance patient safety, satisfaction and systemic sustainability, are becoming increasingly visible [1,2,3,4]. In Dutch maternity care, improving integration and client-centeredness have become central foci in policy and practice, to overcome historically grown systemic fragmentation and lack of responsiveness to clients’ needs and wishes [5,6,7]. Although there is broad agreement on these goals, this is not the case when it comes to how to reach them, and the wide variety of interpretations and initiatives in the field reflect the challenges in daily maternity care practice [8]. Nevertheless, interprofessional and interorganizational collaboration was intensified through the establishment of maternity care networks and experiments with bundled-payment and shared-care-models [7,9]. These new practices were however paralleled by debates in which competing interests and perspectives were accentuated, thus reproducing traditional professional autonomy and hierarchies, and unintendedly reaffirming existing barriers for integration and client-centered practice [7].

Van Kemenade et al. (2022) explain this by pointing out that while the integration of processes and networks was supported, attention to normative integration fell short [10]. They argue that collective reflexivity is needed to fix this flaw [10]. Similarly, others encourage healthcare managers to create reflexive spaces, where professionals and other stakeholders can engage in reflexive dialogues and bridge understanding [3,11]. In accordance with these authors, we understand *reflexivity* as a relational process in which meaning and values are co-constructed, with the potential of social transformation, whereas *reflection* concentrates on individual learning and development [10,12,13]. A growing body of research supports this emphasis on interprofessional learning and reflexivity, showing positive impact on accessibility, coordination, and effectiveness of health services, as well as on professionals’ well-being and patient outcomes and experiences [2,13,14,15,16].

Recent evaluations show that this is also increasingly recognized in Dutch maternity care policy, stating that ‘The hard preconditions for the organization of integrated maternity care are essential, but the development of integrated collaboration starts with the softer side’ [17, p. 28]. In practice, however, healthcare organizations are reticent and struggle with whether and how this ‘softer side’ could be shaped. This particularly applies when it comes to embedding (collective) reflexivity, which requires time and competencies that do not easily fit in the productivity- and efficiency-oriented healthcare system [2,10]. In maternity care specifically, creating reflexive spaces for professionals from across disciplines and organizations is at odds with deep-rooted cultural, organizational, and systemic boundaries, that continue to foster interprofessional and interorganizational distinction and competition rather than collaboration [7,18,19].

In current practice, ‘Perinatal Audits’, in which delegates from all involved professions meet to discuss adverse events in which substandard care is suspected, come closest to such reflexive spaces [20]. It is however debatable to what extent the audits’ structure and focus support openness and reflexivity [21]. Also, exclusively discussing incidents narrows down their learning potential [22]. It is increasingly stressed that this failure-oriented approach to learning, known as Safety-I and dominant in healthcare, would profit from being extended with a Safety-II perspective, which instead positively focuses on learning from how, in everyday practice, things usually go right [22,23]. Moreover, the involvement of patients as integral members of learning healthcare teams is still rare and needs further development [24,25].

Given the above, we wanted to explore the potential of creating reflexive spaces for maternity care professionals and clients, and of applying a Safety-II approach, as a means to promote integration and client-centeredness. Therefore, we initiated the ‘SWING-study’, a Dutch acronym translating into ‘Together towards value-driven integrated maternity care’. We focused on unraveling the following: How do participants experience the process and impact of engaging in reflexive dialogues between professionals and clients from multiple organizations and disciplines in maternity care? What does applying a Safety-II perspective mean for the dynamic of reflexivity and learning? And what potential could reflexive dialogues have for further improving the maternity care system?

## Methods

### Approach

Our approach was novel in multiple ways. First, we united professionals and clients from multiple organizations and disciplines, as we were seeking to balance mutual values, interests, and perspectives and stimulate meaningful, democratic knowledge generation [26,27]. Second, we explicitly focused on initiating reflexive dialogues, instead of on exchanging medical-technical, management, or policy information, as is common in maternity care meetings. Finally, applying a Safety-II approach meant that the dialogues focused on topics and casuistry that represented everyday care practice instead of solely incidents [22,28].

Motivated to spur quality of care, two multidisciplinary and multiorganizational groups of maternity care professionals and clients engaged in the SWING-study. The study was set up as a participatory action research (PAR), to foster empowerment, shared ownership, and impact [2,26]. In PAR, researchers do not play the traditional role of “experts” who “come to collect data”, but rather of facilitators in a shared, non-hierarchical process of reflection, action and learning within a community [29,30]. By creating the right conditions, researchers support awareness growth and dialogically reached mutual understanding, thus strengthening people’s ability to take action and exert influence [26,29,30]. We engaged stakeholders (maternity care professionals, clients and managers from the participating regions) during all stages of the project; from the initial design; in preparing input for the meetings; through intermediary evaluations; until the final evaluation and development of a Toolkit, to share developed tools and lessons learned with the field [31].

### Design & participants

Central in the SWING-study were 22 meetings of two multidisciplinary and multiorganizational groups of maternity care professionals and clients (n = 28) from two regions in the Netherlands (see Table 1). The professionals were recruited via presentations we held in both regions; the clients were invited via these professionals (region X) and a motherboard (region Y). The participating clients were mothers who had given birth in the participating regions within 2 years before the study. All participants self-registered for participation following our verbal and written information about the study aim, consequences of participating, and freedom to withdraw at any time, and signed for their informed consent. Some professionals were allowed by their management to free up paid hours; clients and other professionals invested private time. Furthermore, clients received gift cards and professionals received accreditation points for participating. Work, family, motivational, or health issues sometimes impeded the attendance of meetings; while some participants were almost always present, others attended only occasionally. Overall, the number of participants per meeting ranged from 2 to 10, with an average of 7.3. Thus, representation was sometimes (too) limited, but mostly experienced as sufficient to allow for constructive conversations.

**Table 1:** Participants.


	MEETINGS			INTERVIEWS		
	
	REGION X	REGION Y	TOTAL	REGION X	REGION Y	TOTAL

**Maternity care participants**						

Client	2	1	3	1	1	2

Maternity care assistant	4	1	5	1	1	2

Primary care midwife	3	6	9	3	4	7

General practitioner providing maternity care	0	1	1	0	1	1

Hospital-based midwife	1	2	3	1	2	3

Obstetric nurse	2	2	4	1	2	3

Obstetrician	1	1	2	1	1	2

Pediatrician	1	0	1	1	0	1

***Subtotal***	***14***	***14***	***28***	***9***	***12***	***21***

**External participants**						

Researchers	2*	2*	2*	n/a	n/a	n/a

Chairperson	1	1	2	1	1	2

***Subtotal***	***3***	***3***	***4***	***1***	***1***	***2***

**Total**	17*	17*	32*	**10**	**13**	**23**

* These were the same two researchers in both regions, therefore the total is 32 (and not 17 + 17 = 34)						


In both regions, 11 meetings were organized bi-monthly between 2020–2022, mostly online due to COVID-19-related restrictions. Two independent chairpersons guided the reflexive dialogues, which concentrated on cases from participants’ own practice, further enriched with evidence from previous research and additional data collection within the project (e.g. client meetings, questionnaires). Case selection was done by the participants collectively and informed by the Safety-II approach. Each time, one of the participants would prepare casuistry, aided by guiding questions that we derived from the functional resonance analysis method (FRAM) [32]. The FRAM is a method for graphically modeling (manually or with FRAM-software) workflow in complex socio-technical systems like healthcare, from a Safety-II perspective [32,33]. Instead of searching for (often debatable) causalities, the FRAM focuses on visualizing dependencies, variability, and adaptations in daily (care) practice, and on clarifying differences between ‘work-as-imagined’ (in guidelines and protocols) and ‘work-as-done’ (in practice). During the meetings, we explored whether the FRAM was suitable to support the reflexive dialogues on everyday maternity care practice [33]. We found that participants considered the FRAM as (potentially) valuable for unraveling medical-technical processes, but too time-consuming, complicated, and academic for our meetings. We therefore jointly decided to stop exploring the strict application of the FRAM, although it continued to inspire us when developing materials to support the reflexive process, such as guiding questions to outline casuistry as input for the meetings. Furthermore, we made use of elicitation techniques and materials to trigger creativity and engagement, such as images, objects, film, polls, and (digital) sticky notes.

### Reflexive data collection & analysis

The evolvement of the whole process was logged by the researchers in logbooks, field notes and reports, and the COREQ-checklist helped to guide quality and rigor [34]. Reflexivity was built-in in the research design at multiple levels [35]. Besides reflecting on care practice, participants were also engaged in critically assessing the process itself, during and outside the meetings, collectively and individually, and verbally as well as written (for example through sticky notes, polls, and an evaluative survey during the project). Final evaluative interviews were conducted with individual participants (n = 23), to extend our insight in their personal experiences and validate our observations. Synchronously, ongoing dialogues with regional stakeholders, a project- and steering-group (see Table 2), and our research teams supported the study’s monitoring and reflexive action. Data collection and analysis were thus iterative learning processes of reflective observation, conceptualization and experimentation [36].

**Table 2:** Involved stakeholders and monitors.


	COMPOSITION	ROLE

**Regional stakeholders**	Maternity care professionals as members of regional obstetric partnerships; individual clients and clients organized in a “motherboard”; managers from the participating regions	Periodical meetings to update how the project progressed and/or to give us advice or input and/or to jointly organize meetings (for example a thematic meeting on postpartum care experiences)

**Project group**	Internal researchers, experts, and project managers with backgrounds in midwifery, psychology, sociology, health sciences and public health	Guiding, supervising, and supporting the execution of the study’s activities in daily practice

**Steering group**	External care professionals, researchers and policymakers in midwifery, obstetrics, and client/patient advocacy and public health	Monitoring the project’s development and compliance with the research goals at a more general level, through annual meetings and newsletters


For final analysis in MAXQDA (2022), all reflexive meetings and interviews were audio-recorded, transcribed, and anonymized. Coding was done by the first author, guided through meetings of the research team in which the analysis and possible inconsistencies were discussed. Internship students in midwifery (BSc) and health policy (MSc) also analyzed the data separately, adding analytical triangulation. We first performed reflexive thematic analysis on the interview data, through open, axial, and selective coding, to inductively discover patterns in participants’ experiences with the process and impact of the meetings [27,37]. Subsequently, we analyzed the meetings’ data, zooming in on examples that would either confirm or appear inconsistent with participants’ narratives.

### Navigating engagement & ethics

Our strong engagement in the study undeniably impacted our relationship to the participants, process, and data; a distant approach would have done so too, yet differently. We have strived to outbalance possible bias through the abovementioned ongoing reflexivity and involvement of in- and outsiders to critically assess our methods and analyses. Thereby, this engagement was meaningful for the relationships and trust that developed within the groups, and therefore also a strength.

The study was assessed and approved by the Medical Ethical Review Committee of the VU Medical Center, confirming that the Dutch Medical Research Involving Human Subjects Act (WMO) did not apply (Ref. number 2020.220).

## Results

**Figure 1:**
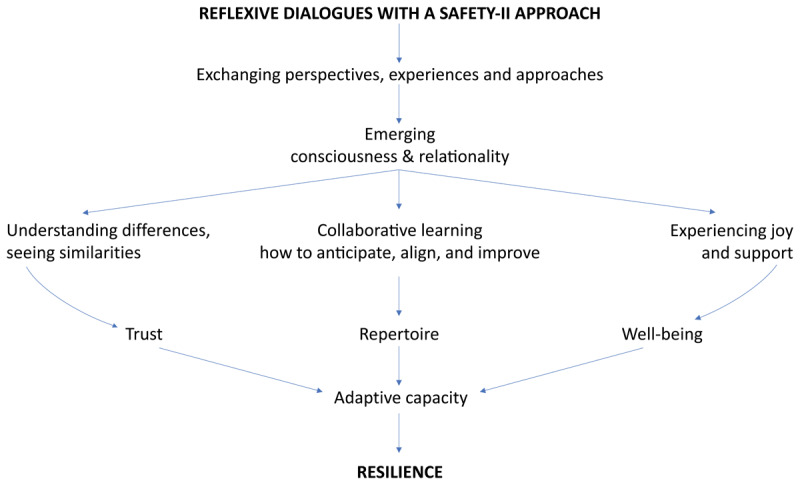
Model of the observed dynamic and impact.

Overall, our findings show how reflexive dialogues with a Safety-II approach increased trust, well-being, and repertoire. We describe our findings along these themes. First, we outline how participants’ improved understanding of mutual differences and similarities contributed to **trust**. Second, we describe how the dialogues impacted their **well-being**, because they were experienced as interesting, enjoyable, and supportive. Third, we show how exchanging perspectives, experiences, and approaches extended participants’ **repertoire**, as it helped them discover new opportunities for anticipating, aligning, and improving. Following this, we provide a case example to illustrate what these reflexive processes looked like in practice. To conclude, we outline how **resilience**, as an overarching theme, summarizes the overall impact of these reflexive dialogues, given the importance of trust, well-being, and repertoire as its enablers. Figure 1 visualizes the dynamic and impact we observed.

### Trust

Above all, participants stressed how the meetings contributed to mutual understanding at an emotional level, and through this, trust. Trust, in turn, is something all participants named a crucial pillar of good collaboration. In the dynamic underlying this building of understanding and trust through the dialogues, three interrelated factors can be distinguished.

The first one is that, through the conversations, participants gained a better ***understanding of existing differences***. Unraveling casuistry step by step, and exchanging viewpoints, helped them to see how each person can perceive and approach care processes differently, depending on one’s knowledge, values, and boundaries, and that all may be equally “right” or “true”. This happened within and between professional groups, as well as between professionals and clients.

*“What I have noticed with this study, is that it is actually quite useful (…) to hear the opinion of such a mother (…) and of the maternity nurse and the obstetrician, who all look at it in a completely different way. (…) When you analyze a case completely during such a conversation (…) you learn to understand each other better. And [you] take that back to daily practice.”*

*Pediatrician*

Second, ***awareness of interpersonal, interdisciplinary, and interorganizational similarities*** also grew through the dialogues. The clients thought it was a revelation to see the “humans behind” the care professionals, who, just like themselves, had personal doubts and struggles.

*“We think doctors are gods (…), they are just humans.”*

*Client*

The professionals learned to see how they, despite often experienced or assumed differences, all grappled with similar dilemmas and barriers in daily care practice. It also made them aware that allocated time for taking a step back to reflect is a prerequisite to allow for such conversations.

*“You’re confirmed in the feeling of ‘Oh ok, I’m not the only one who’s running into it’. (…) Actually every discipline runs into the same problems, and because of that it’s just super meaningful to talk about that with each other, at a different moment than just on the job.”*

*Hospital-based midwife*

Thereby, they stressed that opportunities to connect at a personal level will only become more important, to counterbalance the trend of expanding healthcare networks and merging hospitals, in which collaboration increasingly takes place between people who do not know each other (well).

Third, understanding differences and similarities helped participants realize that, while people may do things you do not like or approve of, their intentions are still good. That awareness, in turn, fostered looking at each other from a perspective of ***leniency, willingness to cooperate, and giving each other space***. This was noted as particularly important in light of larger-scale developments, because paradoxically, discussions on policy, financial and organizational changes regarding the intended integration of maternity care, have often accentuated mutual differences and boundaries instead.

*“When you know each other better you can trust each other better, you know where the other person stands. And you dare … you no longer presume mistrust. Which is quite the case at the moment; when I come in with a client and the obstetrician doesn’t think my policy is quite “top of the bill”, (…) then hostility quickly ensues. Suppose the obstetrician knows me and knows that I always provide good care, then he or she is much more willing to think along.”*

*Primary care midwife*

This dynamic was not limited to the interaction between group-members, but sparked what they called a “*ripple effect*” to their daily care practices, of which one participant gave an imaginative example. Shortly after one of our meetings, which had focused on a midwife who had assisted a family during an unplanned premature homebirth, this pediatrician was herself confronted with a complicated situation. A baby was brought to hospital in a worrisome condition, following a homebirth that she thought would have benefited from earlier medical intervention. As she had experienced in the meetings how much insight hearing *“the perspective of the other, who has been at the beginning of the chain”* could provide, she asked the midwife what had happened. She thus discovered that the midwife had actually timely assessed the severity of the situation, but that the parents had refused to go to hospital, with dramatic consequences.

*“But when you take the time to talk to the midwife like that and hear the whole story, you think ‘Yes, you obviously did recognize that something wasn’t right’.” Pediatrician*

Thanks to this conversation, mutual understanding and compassion emerged, rather than hostility. Although this pediatrician had previously considered discontinuing her participation in the meetings, this incident made her fundamentally reconsider her views on the value of multidisciplinary reflexive dialogues.

However, participating in the meetings was not equally valued and supported by all disciplines, diminishing opportunities for collaborative trust building. Recruiting obstetricians to join the study had been difficult, and the two who joined explained that their obstetric department set other priorities:

*“As a general norm, we don’t free up hours for these kinds of things, because those hours are at the expense of patient care.”*

*Obstetrician*

One therefore gradually withdrew, but the other continued to invest private time, and advocate for embedding multidisciplinary reflexive meetings into standard practice, even though meeting resistance from colleague-obstetricians. (Lack of) systemic incentives further reflected and reinforced such differences in disciplines’ general attitude towards the meetings. Midwives did not get paid hours either but were rewarded with difficult-to-acquire accreditation points for participating in methodical interprofessional meetings. For obstetricians, by contrast, it had been extremely difficult to get their participation in the study’s meetings accredited, and once started, these points proved to be of negligible value to them.

To other participants’ regret, obstetricians were thus not always represented in the dialogues. When present, their input was highly valued. Other participants therefore suggested that, if similar meetings were organized in the future, tailoring casuistry and planning to obstetricians’ interests and schedules would be useful.

*“What I do see is that obstetricians’ attendance in working groups, so where real protocols arise from, is high. (…) Perhaps we could put it in such a form (…) where they feel very much involved.”*

*Primary care midwife*

This tendency to prioritize obstetricians’ needs does however reflect a power imbalance. (Experienced) hierarchies were sometimes also expressed through participants’ wording:

*“**We are** responsible for this, but primary care **is allowed** to do that.”*

*Obstetric nurse*

Although mostly implicit, some participants explicitly mentioned systemic hierarchy:

*“I just like talking to each other about work so to speak. How everyone does it and how everyone stands in it (…) I don’t know if I’m such a big link in it, I mean I’m a maternity care assistant and I do my thing, and apart from that, I don’t have much of a say I guess.”*

*Maternity care assistant*

Thus, while participating in the meetings contributed to collaborative trust-building, seemingly neutral, practical issues like absence and presence could, conversely, contribute to reproducing existing differences and power dynamics.

### Well-being

Besides trust, the dialogues also contributed to participants’ well-being. They particularly valued that the meetings were so different from what they were used to. In daily care practice, they experienced little room for reflexivity. If any, this was mostly limited to monodisciplinary teams and focused on personal development, but much less on group-level functioning, let alone integrated care performance. The multidisciplinary composition of our groups was an important motivator to join the study. In the meetings, participants were especially interested in discussing *why* instead of *how* they exactly did things in daily care practice, by exchanging the motivations and values that supported their (medical-technical) acting and decision-making.

*“I remember an audit where the midwife said, ‘Yes but you’re at home at that moment. You must make a decision. I made this decision then on that basis, and in hindsight, that may have been wrong’. But that subject was ignored, while I think: this is what we should be talking about.”*

*Obstetrician*

The ***Safety-II approach*** was thereby enthusiastically embraced. Learning from everyday care practice instead of adverse events was considered innovative, valuable, and motivating. ‘Perinatal Audits’, although also focusing on multidisciplinary exchange for the purpose of care improvement, were experienced as more judgmental, despite their structured form aimed at supporting psychological safety.

*“What I find difficult is that it’s always about mortality. You’re always searching for culpability, (…) who did what wrong? While that’s actually not the point; the point is: how are we going to improve it?”*

*Primary care midwife*

Besides personal burden, some pointed at possible broader undesirable consequences of this blame culture, like excessive risk-avoidance, medicalization, and health service overuse.

*“In the past, it was only about what went wrong, which I understand, you must learn from that. But… sometimes you really get such a distorted view and then you start acting kind of defensive.”*

*General practitioner providing maternity care*

The positive starting point of our dialogues did not mean that only problem-free care was discussed, but instead, helped participants to feel psychologically safe and challenged to critically reflect on their own and each other’s care practices. Interestingly, even a very experienced chairperson said it challenged him to approach dialogues differently. Participants saw how this provided access to new opportunities for learning and improving, because topics did not necessarily have to regard adverse events. Discussing a complicated case that -despite everything- had ended well, incited the following reflection:

*“I almost regret that it is not an audit, because it is such an instructive case for all care levels.”*

*Primary care midwife*

While the positive impact of the meetings on participants’ mutual relationships and trust aligned with our expectations, we were surprised by them also stressing how they experienced the dialogues as ***enjoyable*** and ***supportive***. Reflecting on casuistry together helped to relieve tension and cope with everyday challenges and adversity, like a form of self-care.

*“The other day a pregnant person of mine almost bled to death. (…) After that you never have any kind of talk: ‘Hey, how was that?’. Or, for example, I had a woman with a dead baby. (…) And then you fall into a kind of hole or something (…) And yes, then I think it’s nice that you can just have a peer conversation about what things do to you.”*

*General practitioner providing maternity care*

They also mentioned how this fostered job and care satisfaction.

*“It’s important to me: my job satisfaction; and you experience plenty of cases and stuff where you doubt (…). And then it’s nice that, occasionally, you have a place where you can discuss: how do you look at it, or: could things be approached differently?”*

*Obstetric nurse*

*“This can help all parties and I think it can reduce complaints in the long run (…) When you engage in a dialogue, you understand where people come from.”*

*Client*

The structured but flexible format of the process further supported this. Although we did ask people to prepare for the meetings and take follow-up actions, there were no immediate, binding obligations. This was felt as sharply contrasting the high pressure and workload in everyday care practice, no matter how passionate they were about their work.

*“This is really one of the few groups, yes actually the only one (…), that energized me, because I didn’t feel like anything was taken from me.”*

*Maternity care assistant/coach*

### Repertoire

On top of contributing to trust and well-being, the dialogues broadened participants’ repertoire, as they discovered new opportunities for aligning, anticipating, and improving. We saw a threefold collaborative learning process. First, reflecting on casuistry heightened participants’ ***consciousness of their personal viewpoints and approaches***. Back in daily care practice, this incited more conscious acting, decision-making, and communication. Seemingly simple insights in decision-making could eventually have systemic implications, like lowering healthcare overuse:

*“Then you start thinking more about what the best care is. And how to approach that.(…) that you engage more in conversations with your patients (…). instead of referring immediately.”*

*General practitioner providing maternity care*

Furthermore, the dialogues gave access to ***learning from alternative perspectives and approaches***, because in the process of reflecting, participants exchanged how they viewed and did things. One person’s standard practice could be another’s new insight:

*“You hear things that make you think, ‘Oh yes, it can indeed be done that way too’.”*

*Primary care midwife*

It made them rethink their habitual ways of doing and experiment with new ideas. The positive experience with mutual learning also made them more tempted to connect with colleagues and ask them for advice or feedback in daily care practice.

*“So then I take it with me and I start thinking about it. (…) As a triage nurse, I always said to clients: ‘Come right over’. But now I think, ‘Gosh, I’m going to ask colleagues what they would do’.”*

*Obstetric nurse*

Third, unraveling casuistry through the dialogues heightened participants’ insight into the consequences of individual behavior and decision-making for the care chain and system. This helped them to ***discover opportunities for anticipating and aligning***, which they took back to daily care practice. For instance, after discussing a case of shared care during birth, in which ambiguity about the division of tasks and responsibilities between a primary care midwife and an obstetric nurse had resulted in conflict, another nurse told how this had prompted her to improve her communicative practices.

*“That you know from each other: what is your responsibility here. Just be much clearer about that. And also put it on paper. That I have it clear for myself and for my colleagues.”*

*Obstetric nurse*

Also, and in defiance of the earlier mentioned debates on integrating care, some conversations uncovered that perspectives on (desired) policy were much more similar than expected.

*Primary care midwife: “I think (…) a lot of complex medical care has shifted from secondary to primary care, including, for example, a very obese woman with a gastric bypass, vitamin deficiencies, and psychological problems as well. (…) I can often tell in advance that those people will become medical anyway. (…) Then we are still half taking care of her, the hospital is half taking care of her, and the lady is going back and forth all the time, not knowing where she stands. (…) And that happens more often, that shared care was agreed, but that I think, ‘I don’t want this, there is a limit to what we can do’.”Obstetric nurse: “The funny thing is [name], that I just thought (…) you guys wanted to keep hold of clients as much as possible.”*

Evaluating the project, months later, this nurse still recalled that conversation being “*an eye-opener”*.

Participants also reflected on the broader structures in which work is done. Besides interpersonal differences, existing policy differences between professional groups and organizations were repeatedly addressed as a source of variability.

*“Then those protocols turned out not to be the same at all. I had never realized (…) that as a midwife, you don’t know in advance which hospital you’re going to and whether there’s room, and that you actually have to know from each hospital what the protocol is there. So I found that very clear and insightful.”*

*Obstetrician*

Given that many had previously been unaware of these differences, the dialogues explicitly helped to reveal potential for further policy alignment. Taken together, the meetings uncovered numerous opportunities for alignment and improvement at the interpersonal, interdisciplinary, and interorganizational level. However, sharing insights and potential action points more broadly, to initiate action and change throughout the organization, remained difficult; even further exacerbated by the restrictive measures due to COVID-19. This difficulty especially applied to the region characterized by a larger size and complexity on the one hand, and less developed organizational structure on the other hand. In the smaller region, it was clearer to which persons or working groups professionals could turn to share ideas. However, expansion due to hospital mergers also happened there, and overall, we saw that participants experienced little power to create impact, causing frustration and fatigue.

*“It just takes a lot of time and sometimes it’s like flogging a dead horse.”*

*Obstetric nurse*

Interestingly, this was the same nurse who had improved her communicative practices; impact that she had not recognized as such herself, but very relevant to quality of care. Moreover, while explicit action points were not always (immediately) followed-up, things that had not been framed as such could also incite further reflection or action. Implicit small changes happened throughout the project, but barely visible for the participants. Our involvement was important to help uncover this learning dynamic.

### Case example

To make tangible what these reflexive processes looked like in practice and how they can yield learnings for quality improvement in maternity care, Box 1 illustrates how participants proceeded from reflection to action over the course of four meetings:

**BOX 1: FROM INSIGHT TO IMPACT**

**§SESSION 1:**

As input, the researchers prepared casuistry by interviewing two women who had been referred from home to hospital during labor, due to stagnating dilatation. While the former had felt saddened and disempowered, the latter had experienced the exact opposite. In the session, the group mirrored the two cases and concluded that (lack of) experienced autonomy had been a key determinant of these differing experiences. This, in turn, had been strongly impacted by differing communicative and shared decision-making (SDM) practices.

**§SESSION 2:**

We therefore subsequently zoomed in on communication and SDM specifically. Participants reflected on their own and each other’s styles, practices, and values, which made them realize that they tended to explain and advise standard care procedures (mostly a single one), to which people would generally consent, instead of supporting decision-making based on a variety of options.

*“We inform the patient and name what we are going to do and what it entails. But actually, that is barely giving choice, you just explain. Then of course they say: ‘Oh well, fine’.*

*”Hospital-based midwife*

**§SESSION 3:**

As a preparation, participants individually reflected on what elements they thought were conditional for having a constructive conversation with clients to support SDM. During the meeting, participants mirrored their knowledge and practices with each other and with existing research evidence on this topic.

While the professionals’ conversation accentuated the need to give people *more* choice, the client added further nuance by stressing their role in supporting *better* decision-making. She thought care professionals tended to overestimate clients’ assertiveness. For parents, having a baby is a rare and often overwhelming experience, and asking questions or speaking up can be very challenging.

*“I took a decent pregnancy course, with all the ‘ask questions’-tips, and I was completely prepared. I was ready. But after 33 hours of labor, I really didn’t have the presence of mind to say: ‘Wait a minute, just calmly explain to me why (…) this is necessary’.”*

*Client*

Her input highlighted that, besides knowing and understanding factual information, a relational process is required for clients’ agency to flourish. This dialogue helped the participants discover alternative ways of communicating and supporting SDM in daily practice, which they started to explore following the meeting.

**§SESSION 4:**

In the last meeting on this topic, participants reflected on how they had applied new insights in daily care practice, with positive results.

*“I now try to include this as a standard element, at the very beginning of the pregnancy (…). I explain about freedom of choice (…), and that the first option is always not to do something, and that people always have their own choice. (…) Then sometimes people come up with experiences in which they felt they had no choice. So, yes, I do get positive reactions to that.”*

*Primary care midwife*

The awareness that ‘doing nothing’ is always one of the options, had concrete impact:

*“Last week I had done an* external version*, which didn’t succeed, even though I felt that it should be possible. And I always counsel very positively about doing a second attempt. But then I saw that patient again and I suddenly tasted that maybe she didn’t want that after all. And then I really had to pause for a moment, thinking, ‘Oh no, we have to go back to what you want, and not to what I think is good, or possible’. (…) And when I mentioned that she did indeed say that she felt it might have been good enough.”*

*Obstetrician*

Participants realized that this urge to act is rooted in healthcare professionals’ culture:

*“We have become doctors or caregivers to do something for people, but doing nothing can sometimes also be a lot.”*

*Hospital-based midwife*

Other systemic boundaries that the dialogues brought forward were time constraints that limit opportunities for meaningful conversations on choice; an especially pressing issue given growing staff shortages. Fear of being held accountable for not sticking to guidelines, albeit on clients’ explicit request, was also mentioned as limiting choice.

### Resilience

In summary, our findings show how these reflexive dialogues with a Safety-II approach increased trust, well-being, and repertoire. We found this through the inductive analysis and argued these might be building blocks for resilience, but wanted to check whether this aligned with the literature. This confirmed that trust, well-being, and repertoire are essential enablers of and resources for adaptive capacity, which is a key characteristic of resilience [38,39]. Also, evidence for collaborative learning within and across system levels being fundamental to the enactment of resilience in healthcare, supports the understanding of the dialogues as its promoters [38,40]. We therefore conclude that ***resilience***, as an overarching theme, summarizes the overall impact of the reflexive dialogues. Resilience does not refer to a state of being, but to how an entity performs; maintaining operability under variable conditions and responding through continuous anticipation, adaptation, learning, and transformation [23,41]. The dynamic of reflexivity and learning we observed throughout the reflexive dialogues strongly resonates this growing capacity.

Evaluating the project, almost all participants articulated the wish to continue and embed multidisciplinary and multiorganizational reflexive meetings as standard practice in maternity care. However, they were familiar with healthcare management’s hesitance to invest by allocating (paid) working hours and budget. Participants stressed that maternity care as a whole would benefit from taking the value of meetings like these more seriously.

*“It’s just a huge investment in collaboration. And when there is better collaboration, outcomes are automatically better (…) you can’t measure that of course, but it is.”*

*Primary care midwife*

*“We had a heated discussion about it. Because well, doctors are particularly interested in* *RCTs**. One of my colleagues said: ‘It may not be a sexy RCT but this is also of essential importance’. (…) For job satisfaction also. And job satisfaction is very important when it comes to downtime, illness, and the like. And thus, ultimately, just for all of maternity care.”*

*Hospital-based midwife*

## Discussion

To promote integration and client-centeredness, we explored the potential of creating reflexive spaces in maternity care. We found that reflexive dialogues between care professionals and clients from multiple organizations and disciplines contributed to growing trust, well-being, and expanded repertoire. Moreover, we saw important enablers and resources for resilience, particularly adaptive capacity and collaborative learning. Thereby, the Safety-II approach acted as a facilitator, by positively framing the dialogues, even when experienced difficulties were discussed. We therefore conclude that the overall impact of Safety-II guided reflexive dialogues is that they foster resilience in maternity care.

Amplifying resilience is considered necessary to further improve the complex adaptive system that healthcare has become [1,28,42,43]. This will only become more urgent given yet unknown but foreseeable crises and disasters that will affect future healthcare indirectly (climate change) or directly (pandemics) [41,44]. The importance of reflexive learning for promoting resilience has previously been articulated, but was little embraced in healthcare practice [3,40,44]. One reason may be the lack of examples of actionable interventions showing positive impact [15,41,45]. Our study contributes to filling this gap. The finding that the reflexive dialogues impacted participants’ personal practices, their mutual alignment, and offered insights into broader systemic structures and opportunities for improvement, shows their potential at multiple levels.

The well-being aspect of the impact we found deserves special attention. While policy and practice to improve and integrate healthcare increasingly acknowledge clients’ positive experiences as valuable outcomes, adequately supporting healthcare professionals is often overlooked [46,47,48,49]. Meanwhile, developing the envisioned systemic reforms is largely left to individual professionals in the field, again putting increasing demands on them, on top of healthcare-broad challenges such as staff shortage, bureaucratization, and healthcare work’s inherent stress risks [7,49,50,51]. This translates back into high absenteeism, turnover, and outflow, reinforcing pressure on the already stretched workforce [4,50,52].

Leadership and governance are needed to break this vicious cycle [39,47,52]. Facilitating reflexive spaces is one step leaders could take to better support and protect healthcare professionals. This would include allowing personnel to free up hours for participating and providing practical support (organization, chairpersonship, reporting etc.) [31]. Although this may seem counterproductive given workforce shortages, diminishing returns of traditional approaches underscore the need for finding new ways to spark improvement. Reflexive dialogues between care professionals and clients serve multiple goals on the policy agenda, simultaneously fostering interprofessional and interorganizational alignment, client-centeredness, and workforce well-being [3,17,42,49,52].

Overall, leaders could guide the change process more positively. Disputes at administrative levels, especially between the professional associations of midwives an obstetricians, continue to highlight mutual differences and affect collaboration at local levels [7,53]. “Losses” coming with integration, and the need to safeguard autonomy, are thereby often emphasized [8,54]. Although the importance of autonomy is undebated, it highlights but one side of the coin [55,56]. People do not experience autonomy in isolation, but in relation to each other. Our study reaffirms how relationality, in particular, can be a resource for trust, well-being and repertoire, and thus resilience [55]. Better communicating these gains would not only be more motivating, but also elucidate how the schism between autonomy and integration is actually artificial, since trust and relationality are indispensable for giving each other space, and thus autonomy.

Reflexive spaces alone will not solve the challenges maternity care is facing, but no “solution” will; complex, multifactorial problems demand multifaceted approaches to tackle them [57]. Systemic transformations are long-term, complex processes, in which micro-experiments can trigger habitualization to new practices as part of a broader strategy [2,58]. This study offers a practical example of how healthcare leaders can help create the conditions for integrated, relation-centered care, starting today.

### Strengths & limitations

A strength of this study is that we included two maternity care regions that differed greatly in size, population, and organizational structure. Also, the diverse composition of both groups, and especially the inclusion of clients, was innovative [24,25]. A limitation is however that the impact we found is based on our observations of the dialogues and on what participants self-reported. Pairing observations with evaluative interviews did prove helpful for understanding and cross-checking dynamic and impact. Complementing this approach with observations in daily care practice could be valuable -although methodologically challenging- for further validation.

Furthermore, the small scale of the study limits its impact, as did the difficulties we encountered regarding communicating results more broadly within the regions. While already challenging under normal circumstances, as participants’ experiences illustrated in the results section, COVID-19 constraints further exacerbated this. Meetings were mostly canceled, and informal encounters diminished, evaporating opportunities to share insights with non-participants. Thereby our role as initiators and communicators unintendedly remained more dominant than envisioned [26]. Workload and demoralization made participants reluctant to engage further. Impact might have benefited from power and ownership being more equally shared by participants [26]. Also, more physical presence within the participating regions could have facilitated the building of relationships and learning networks, and thereby impact. During live meetings, conversations progressed more fluidly, and participants valued the opportunity for informal interaction. However, meeting online did not withhold them from expressing criticism or vulnerability, and lowered practical barriers for participating.

## Conclusion

This study shows how Safety-II guided reflexive dialogues between professionals and clients from multiple organizations and disciplines foster trust, well-being, and repertoire, and through this, resilience in maternity care. By structurally stimulating, facilitating, and embedding reflexive spaces, healthcare leaders could reinforce the transformation towards an integrated, relation-centered maternity care system.
